# A Survey of the *Agrotis* of Iran

**DOI:** 10.1093/jis/14.1.95

**Published:** 2014-07-18

**Authors:** Sh. Feizpoor, A. Shirvani, M. Rashki

**Affiliations:** 1 Young Researchers Society, Shahid Bahonar University of Kerman, Kerman, Iran; 2 Department of Plant Protection, Faculty of Agriculture, Shahid Bahonar University of Kerman, 76169-133 Kerman, Iran; 3 Department of Biodiversity, Institute of Science and High Technology and Environmental Sciences, Graduate University of Advanced Technology, Kerman, Iran

**Keywords:** Agrotini, fauna, Noctuinae, systematics

## Abstract

The present study reviews the genus *Agrotis* Ochsenheimer, 1816 (Lepidoptera: Noctuidae: Noctuinae) in Iran from a taxonomic and faunistic point of view. An identification key of external features is presented for 16 Iranian species and subspecies. A description of each taxon is presented based on external male and female genital characteristics. Diagnostic features and comparisons with the closest relatives are given for each species. Original combination and citation with the synonymy of each species or subspecies are expounded as well as their distribution and bionomy. Adult moths and male genitalia are illustrated.

## Introduction

The territory of Iran primarily encompasses semi-desert plains. However, it also is covered by two mountain chains (i.e., Elburz and Zag-ros) with xeromountain type ( Varga 1996 ) vegetation. Despite the rather dry climate of the country, the northern line of the country comprises humid forests. Owing to its various geographic and climatologic diversity, relatively rich fauna and flora are expected in the country.

Cutworms, members of the genus *Agrotis* Ochsenheimer, 1816 (Noctuinae: Agrotini), feed on roots and foliage of their host plants, and many species are important pests of crops. Some of the larvae spend the day inside the soil and consume leaves at night. *Agrotis* spp. inhabit various geographical regions and prefer open habitats. The prominent feature of the genus is the pectinate antennae of the male as well as the configuration of male and female genitalia (described by Fibiger 1990 ), especially the elongated vesica (the longest in the subfamily) with basal and apical swellings and basal diverticulum with a basal scobinated belt. A very long appendix bursae (sometimes five to six times longer than corpus bursae), fitted with the vesica of the male, is a unique feature among *Agrotis* species. These particular structures encouraged Fibiger (1990) to propose a new method to delimit the species of the genus based on measuring the vesica and appendix bursae length of both male and female sexes.

Until now, many researchers have primarily studied this genus from the faunistic and taxonomic point of view (e.g., Wiltshire 1975 , 1994; Fibiger 1990 , 1993, 1997; Hacker 1990 ; Ivinskis and Miatleuski 1999 ; Kravchenko et al. 2006 ; Michael 2006 ; Lafontaine and Schmidt 2010 ). To date, 103 species of this genus have been described worldwide. Of those, 80 species exist in the Palaearctic region. Twenty-three *Agrotis* species of are endemic to North America in the Nearctic region and do not exist in the Palaearctic region ( Lafontaine and Schmidt 2010 ). The European fauna, with 23 species, have been well studied by Fibiger (1990 , 1993, 1997), who classified these species into 10 species groups: the *fatid-ica,* the *cinerea,* the *graslini,* the *segetum,* the *endogaea,* the *vestigialis,* the *ripae,* the *trux,* the *crassa,* and the *biconica.* The Near East and its neighboring areas contains around 38 *Agrotis* species ( Hacker 1990 ; Wiltshire 1994 ; Ivinskis and Miatleuski 1999 ). Among them, Iran is represented by 16 species, Turkey by 19 species ( Kemal et al. 2007 ; Koçak et al. 2008 ), Israel by 19 species ( Kravchenko et al. 2006 ), Turkmenistan by six species ( Ivinskis and Miatleuski 1999 ), and Cyprus by eight species ( Fibiger et al. 1999 ).

The 16 Iranian species are arranged into six species groups: *cinerea, segetum, endogaea, trux, bigramma,* and *spinifera (A. crassa* ([Hübner], 1803) and *A. biconica* Kollar, 1844 are synonyms of *A bigramma* (Esper, [1790]) and *A spinifera* (Hübner, [1808]), respectively, so in this study their groups are treated as synonyms of the *bigramma* and *spinifera* species groups). The *cinerea* species group, with Southwestern Palaearctic distribution ( Bond and Gittings 2008 ), is represented in Iran by one species, *A. cinerea* ([Denis & Schiffermüller], 1775), which is distributed in western Iran ( Ebert and Hacker 2002 ). *A. segetum* ([Denis & Schiffermüller], 1775), a member of the *segetum* species group, lives in all geographical regions in Iran in spite of its other congroup, *A. clavis* (Hufnagel, 1766), which populates the temperate zones in the north and northeast parts of the country. In Iran, three species belonging to the *endogaea* species-group *(A. herzogi* Rebel, 1911, *A. puta* (Hübner, 1803), and *A. sardzeana*Brandt, 1941 ) are dispersed in the warm and dry lands in the southern parts of the country ( Ebert and Hacker 2002 ), the preferred habitat mentioned by Fibiger (1990) . *A. puta* have also been reported from the European countries ( Bond and Gittings 2008 ). The cosmopolitan species ( Fibiger 1997 ) *A. ipsilon* (Hufnagel, 1766), *A. exclamationis* (L., 1758), and *A. trux* (Hübner, [1824]) comprise the *trux* species group and are distributed in all climatic regions. Due to the lack of comprehensive faunistic investigations, the distribution pattern of some species is based on local surveys. The significant example is the *bigramma* species group, in which *A. bifurca grossi* Hacker & Kuhna, 1986 is reported from the east ( Ebert and Hacker 2002 ) and *A. obesa scytha* Alphéraky, 1889 and *A. bigramma* occupy northern latitudes ( Wieser and Stangelmaier 2005 ), although a few specimens of *A. obesa scytha* have been locally collected by the authors from the southern part of the country. The taxonomic placement of the sixth species group, *spinifera* , is dubious; therefore, this survey follows Fibiger (1997) , in which *A. spinifera* , *A. lasserrei* (Oberthür, 1881), *A. benigna* (Corti, 1926) and *A. psammocharis* Boursin, 1950 are referred as members of the *spinifera* species group. All members are locally distributed in both semiarid and temperate regions ( Ebert and Hacker 2002 ; Wieser and Stangelmaier 2005 ; Kravchenko et al. 2007 ).

The present paper reviews 16 species of the genus *Agrotis* in Iran. Citations of the original combination, synonymy, and distribution of each species are given. Adult moths and their genitalia are described; diagnostic features, bionomy, and notes on their distribution are discussed. An dentification key is presented based on the external characteristics. Adult moths and their genitalia, if they are presented, are illustrated.

## Materials and Methods

Main data of the recorded noctuid moths were obtained from a bibliographic survey (e.g., Hacker 1990 ; Ebert and Hacker 2002 ). Furthermore, the results of recent expeditions carried out in different parts of the country (Kerman, Sistan va Balouchestan, Semnan, Markazi, Ardabil, Fars, Golestan, Khorasan, Hormozgan, and Esfahan provinces), were added. Distinctive regions included diverse vegetation, and geographical locations were chosen and sampled in separate provinces beginning in 2004. Two kinds of light traps (portable and constant) applied to collect material and were sampled regularly. Chloroform solution was used to kill the attracted moths inside the light traps. The collected moths were maintained with insect pins on spreading boards. In order precisely identify the species, the genitalia of moths were extracted and mounted using Euparal mounting medium. The adult and genitalia images were taken by a Canon Digital camera (model Power Shot A710, www.canon.com ). Terminology for genital structures and wing morphology follows that of Fibiger (1977 , 1990). All collected and studied material (except those of *A. lasserrei* and *A. cinerea,* which are deposited in the Hungarian Natural History Museum) are deposited in the Collection of Entomology, Shahid Bahonar University of Kerman, Iran.

## Taxonomic Results

### Identification key based on external features

1. Noctuid maculation conspicuous, sharply defined………………………………………2 1 **'** . Noctuid maculation obsolescent………..13

2. Subterminal area and medio-distal part of the reniform stigma with black dart shaped marks………………………………….. *ipsilon* 2 **'***.* Three dart shaped marks absent…………3

3. Claviform stigma dark, elongated………...4 3 **'** . Claviform stigma small or medium……...8

4. A dark patch present between the orbicular and reniform stigmata……………………….5 4 **'** . Dark patch absent………………………..7

5. Claviform stigma sharply defined, pointed, pure black, crosslines obsolescent *spinifera* 5'. Claviform stigma fade, brownish, crosslines present ………………………………...6

6. Forewing veins well outlined by light scales, hindwings dark brown in outer half……………………………… *obese scythe* 6'. Forewing veins simple, hindwings whit ish…………………………………….. *bifurca*

7. Orbicular stigma elongate, thin, connected to reniform stigma………………… *sardzeana* 7 **'** Orbicular stigma globular, free from reniform stigma……………………….. *bigramma*

8. Orbicular stigma elongate, thin, spindle shaped, whitish, centered with dark spot……9 8 **'** . Orbicular stigma globular………………11

9. Crosslines well defined, sharp, dentate …………………………………... *lasserrei 9 **'** .* Crosslines obsolescent………………….10

10. Forewing unicolor, dark gray……. *herzogi* 10'. Forewing whitish cream, basal area dark, terminal area with conspicuous brown patch medially………………………………… *.puta*

11. Patagia with dark line, forewing unicolor, stigmata filled with black……... *exclamationis* 11ʹ. Patagia without dark line, at least one stigma as ground color……………………..12

12. Forewing dark brown, crosslines well defined, double, hindwing brownish especially at outer margin, discal spot present……. *clavis* 12ʹ. Forewing beige, costal margin with dark spot apically, hindwing whitish………….. *trux* 12ʹʹ. Forewing beige to dark gray-brown, costal margin without apical spot………. *segetum*

13. Claviform stigma missing……………...14 13ʹ. Claviform stigma present………. *benigna*

14. Orbicular stigma always present, antemedian and postmedian lines obsolescent…………………… *psammocharis* 14ʹ. Orbicular stigma present or absent, antemedian and postmedian lines distinct... *cinerea*

## The checklist

### Genus: *Agrotis* Ochsenheimer, 1816

*Agrotis* Ochsenheimer, 1816, *Die Schmetterlinge von Europa***4** : 66. Type species: *Noctua segetum* [Denis & Schiffermüller], 1775. L.t.: Austria, Vienna district.

[Synonymy: *Agrotis* Hübner, 1806 [rejected name]; *Scotia* Hübner, 1821; *Georyx* Hübner, 1821; *Agronoma* Hübner, 1821; *Noctua* Boisduval, 1828; *Psammophila* Stephens, 1850; *Lycophorus* Staudinger, 1901; *Powellinia* Oberthür, 1912]

## The *cinerea* species-group

### *Agrotis cinerea* ([Denis & Schiffermüller], 1775)

*Phalaena* ( *Noctua* ) *cinerea* [Denis & Schiffermüller], 1775, *Ankundung Eines Systematischen Werkes von den schmetterlinge der Wienergegend***80** . L.t.: Austria, Vienna district. [Synonymy: *denticulatus* (Haworth, 1803); *tephrina* Staudinger, 1901]

**General distribution.** Southwestern Palaearctic ( Bond and Gittings 2008 ). Caucasus, Armenia, Turkey ( Hacker 1990 ), Iraq, Levante, Morocco, Tunisia, Libya, Southern and Central Europe ( Kravchenko et al. 2007 ).

**Distribution in Iran.** Lorestan Province, Elburz Mountain ( Ebert and Hacker 2002 ).

**Fig 1.**
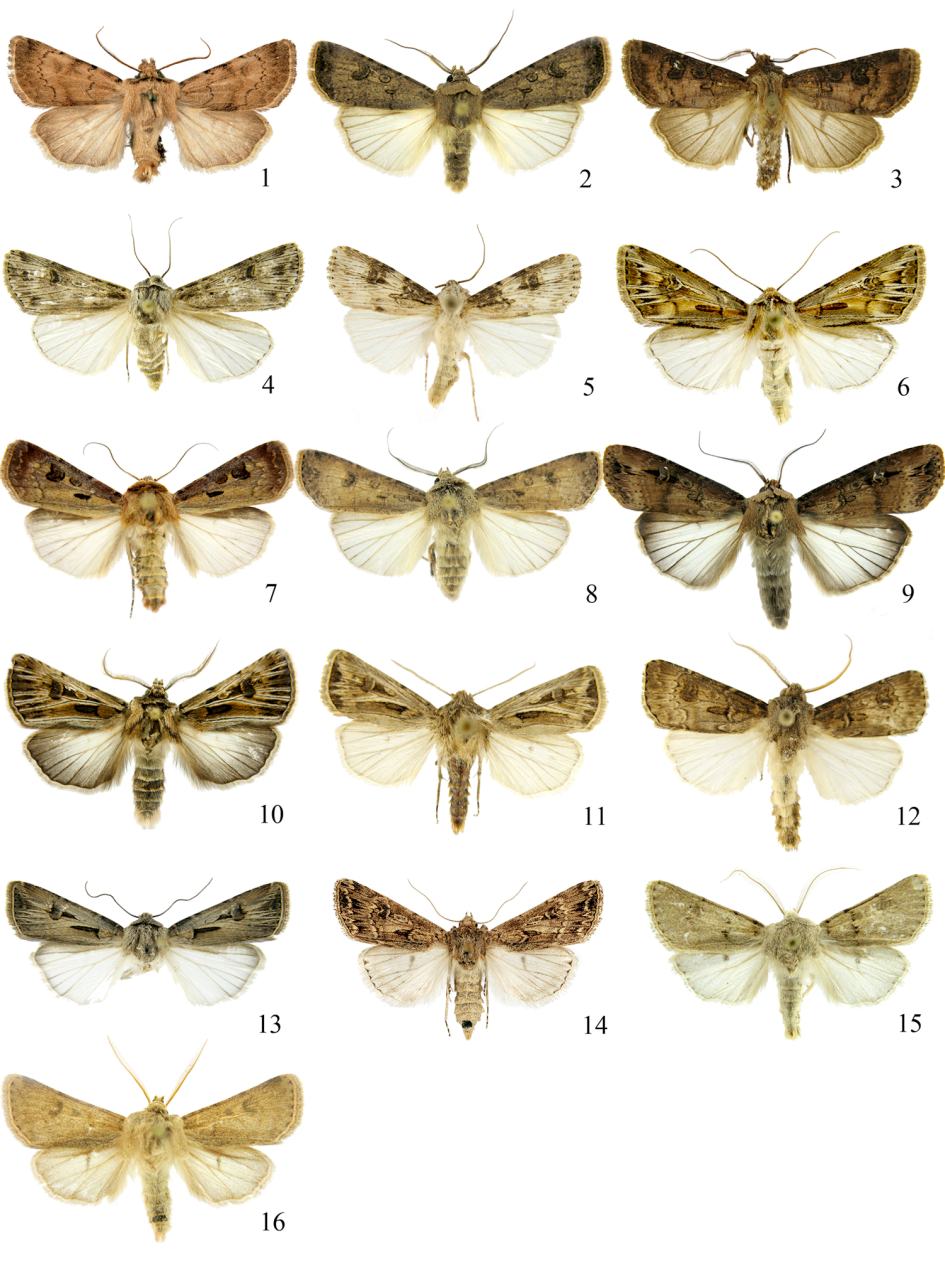
Male *Agrotis* spp. 1. *A. cinerea; 2. A. segetum;* 3. *A. clavis;* 4. *A. herzogi;* 5. *A. puta;* 6. *A. sardzeana; 7. A. exclamationis;* 8. *A. trux;* 9. *A. ipsilon;* 10. *A. obesa scytha;* 1 1. *A. bifurca;* 1 2. *A. bigramma;* 13. *A. spinifera;* 14. *A. lasserrei* ; 1 5. *A. benigna* ; 1 6. *A. psammocharis.* High quality figures are available online.

**Description.** Male ( Figure 1 ). Antennae of the male relatively pectinated. Wingspan 33–40 mm; forewing light brown to light gray, reniform and orbicular stigmata distinguishable, claviform stigma missing, crosslines lines distinct, crenate; hindwings light cream with discal spot. Female generally darker, antennae filiform.

**Male genitalia.** Described by Fibiger (1997 , Figure 57). Costal margin slightly arched medially, cucullus slightly broader than sacculus, basally rounded, clasper thickened. Vesica three times as long as the aedeagus, basal swelling small, apical swelling present.

**Fig 2.**
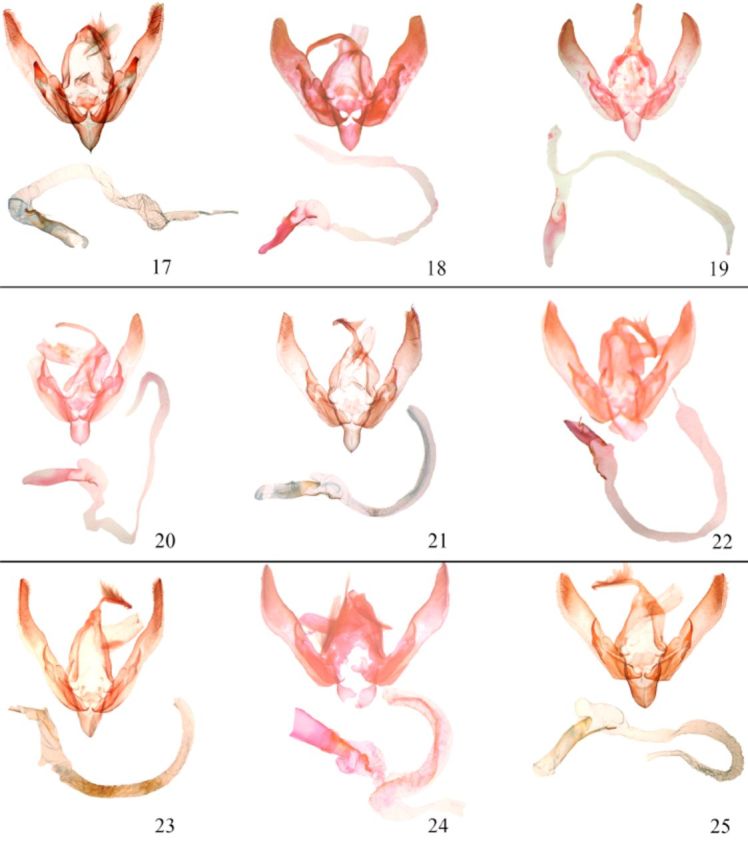
Male genitalia (armature and vesica) of *Agrotis* spp. 17. *A. segetum* ; 18. *A. clavis* ; 19. *A. herzogi* ; 20. *A. puta* ; 21. *A. sardzeana* ; 22. *A. exclamationis* ; 23. *A. trux* ; 24. *A. ipsilon* ; 25. *A. obese scytha.* High quality figures are available online.

**Female genitalia.** Described by Fibiger (1997 , Figure 57). Ovipositor short, anterior apophyses expanded anteriorly; appendix bursae about more than two times longer than corpus bursae; corpus bursae drop-like.

**Fig 3.**
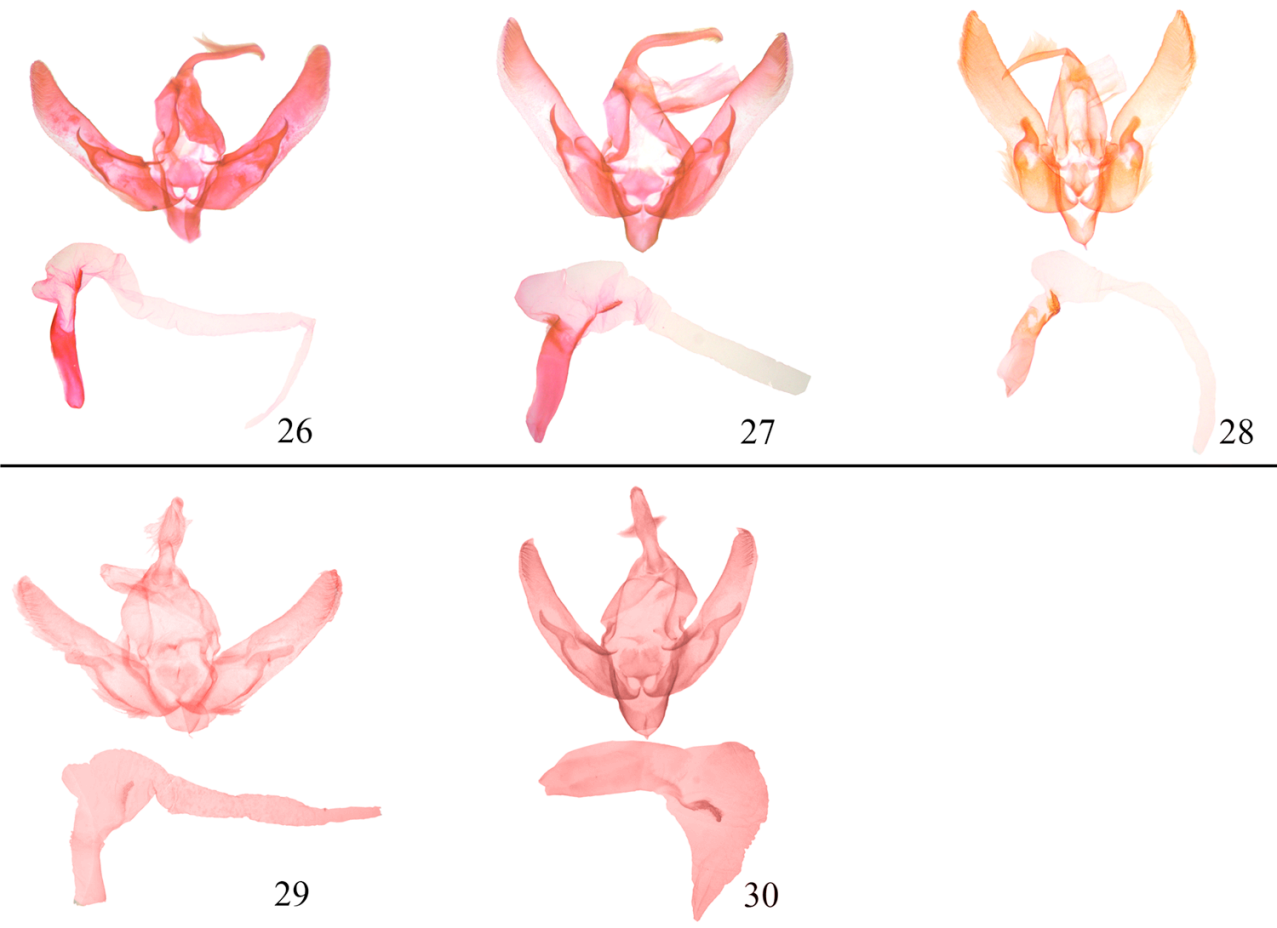
Male genitalia (armature and vesica) of *Agrotis* spp. 26. *A. bifurca* ; 27. *A. bigramma* ; 28. *A. spinifera* ; 29. *A. benigna* ; 30. *A. psammocharis* . High quality figures are available online.

**Bionomy.** Univoltine. Flight period of the moths is May, June, and July. Adults are attracted to light, flowers, and sugar. It inhabits open areas covered by shrubs or scarce trees. The early stages feed on herbaceous and short-term growth plants, including Lamiaceae, Caryophyllaceae, Fabaceae, and Polygonaceae families.

**Taxonomic notes.** Its external and genital characteristics are quite distinguishable; grayish ground color, missing claviform stigma, weakly defined crosslines; short valve in the male genitalia, with rounded cucullus and long clasper.

**Material examined.** 1 ♂, Macedonia, Petrina plan. Leg. R. Lunak.

## The *segetum* species-group

### *Agrotis segetum* ([Denis & Schiffermüller], 1775)

*Noctua segetum* [Denis & Schiffermüller], 1775, *Ankundung eines systematischen werkes von den Schmetterlinge der Wienergegend***81** . L.t.: Austria, Vienna district.

[Synonymy: *spinula* sensu Donovan, 1801; *catenatus* (Haworth, 1803); *pectinatus* (Haworth, 1803); *monileus* (Haworth, 1803); *connexus* (Haworth, 1803); *corticea* sensu Haworth, 1803; *corticcus* misspelling; *subat-ratus* (Haworth, 1803); *nigricornatus* (Haworth, 1803)]

**General distribution.** Palaearctic region, Africa, and Oriental regions including Sinai (Wiltshire 1948); Turkmenistan, Lebanon, Syria, Iraq, Israel, Afghanistan, Russia, Turkey, Armenia, Caucasus, Egypt ( Hacker 1990 ); Cyprus ( Fibiger et al. 1999 ); Mongolia ( Gyulai and Ronkay 1999 ); Jordan ( Hacker 2001 ).

**Distribution in Iran.** It is widespread almost in all climatic regions in Iran. Guilan, Tehran, Elburz Mountains, Azarbayjan-e-gharbi, Khusestan, Bushehr, Hormozgan ( Ebert and Hacker 2002 ); Kerman, Khorasan, Semnan.

**Description.** Male ( Figure 2 ). Antennae strongly pectinated basally. Wingspan 36–39 mm; the forewing coloration different, noctuid maculation complete, darker than ground color, antemedian and postmedian lines distinguishable, subterminal line distinct, sub-terminal area dark brown; hindwings pearl to white. Female like male, except antennae filiform.

**Fig 4.**
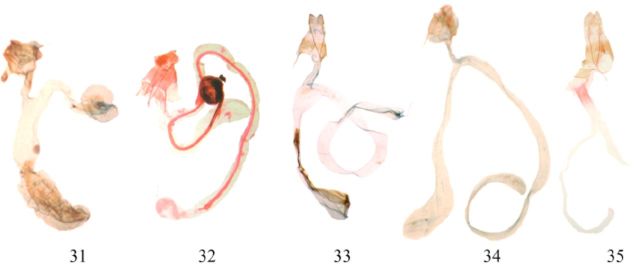
Female genitalia of *Agrotis* spp. 31. *A. segetum* ; 32. *A. herzogi* ; 33. *A. sardzeana* ; 34. *A. ipsilon* ; 35. *A. spinifera* . High quality figures are available online.

**Male genitalia** ( Figure 17 ). Valve long, pointed, clasper short, thickened. Aedeagus tubular; vesica tubular, more than four times as long as the aedeagus, wrinkled, basal diverticulum small, scobinated bar bent, in moderate size, apical swelling cylindrical. **Female genitalia** ( Figure 31 ). Ovipositor short; Ductus bursae somewhat sclerotized; appendix bursae curved, slightly longer than corpus bursae; corpus bursae two times as broad as the appendix bursae.

**Bionomy.** Multi vol tine. The moths flying period is late winter to early fall. They are usually attracted to light and occur in every location from forest to desert habitats in varied altitudes. Larvae are the most important pests in cereals and vegetables.

**Taxonomic notes.** This species can be confused with *A. trux,* but antennae of the male are less pectinated and apical sign on the costal margin of the forewing is more distinct in *A. trux.*

**Material examined.** 7 ♂ *,* 5 **♀** , Iran. Prov. Tehran, Tarbiat Modares University, College of Agriculture, 09. 2005. leg. A. Shirvani; Prov. Kerman, Honouj, 19.03.2004 and 21.03.2006. leg. A. Shirvani; Prov. Kerman, Lalezar, 3200 m., 29°27'33"N 56°48'33"E, 25.06.2009. leg. M. Shoghali.

### *Agrotis clavis* (Hufnagel, 1766)

*Phalaena clavis* Hufnagel, 1766, *Berlin. Mag.***2** : 426. [Synonymy: *corticea* (Denis & Schiffermüller, 1775); *clavigerus* (Haworth, 1803); *subfuscus* (Haworth, 1803)]

**General distribution.** Eurasiatic. In the temperate zones, eastwards to Korea and Northeast China ( Fibiger 1990 ).

**Distribution in Iran:** Ardabil.

**Description.** Male ( Figure 3 ). Antennae strongly pectinated, towards the tip this feature decreases. Wingspan 34–41 mm; forewings brown to dark brown, thorax slightly brighter, noctuid maculation complete, reniform and orbicular stigmata large, dark brown surrounded with black line, claviform stigma lighter than the other ones, cross lines crenate, subterminal line obsolescent; hindwing light cream mixed with light gray, discal spot present. Female similar to male, antennae filiform.

**Male genitalia** ( Figure 18 ). Valve nearly quadrangular terminally, costal margin medially arched, clasper thickened. Aedeagus tubular; vesica tubular, more than six times as long as the aedeagus, basal swelling multi-pouched, slightly broader than the aedeagus, scobinated bar of moderate size.

**Female genitalia.** Described by Fibiger (1997 , Figure 63). Ovipositor short, posterior and anterior apophyses short as long as ductus bursae; appendix bursae tubular, nearly two times longer than corpus bursae; corpus bursae drop-shaped.

**Bionomy.** Univoltine. Adults are on the wing in June and July and are attracted to sugar and light ( Fibiger 1990 ). Occurs in various altitudes. Larvae feed on the leaves and roots of herbaceous plants.

**Taxonomic notes.** The adult moths could be confused with *A. segetum* , *A. exclamationis* , or *A. trux* , but *A. segetum* have a whitish hindwing. Adult *A. exclamationis* have a black line on their patagia, and in *A. trux* a distinct blackish costal mark is presented at the apex of the forewing ( Fibiger 1990 ).

**Material examined.1** ♂ , Osterreich, Heili-genblut, 21.08.1964, Dr. Ehik Gy.

## The *endogaea* species-group

### *Agrotis herzogi* Rebel, 1911

*Agrotis haifae herzogi* Rebel, 1911, *Verh. Zool.-Botan. Ges. Wien***61** : (143). L.t.: Lebanon.

[Synonymy: *hoggari* Rothschild, 1920; *minima* Turati, 1924; *securifera* Turati, 1924; *saracenica* Tams, 1925; *leroyi* Lucas, 1940]

**General Distribution.** North Africa, Arabian countries toward Iran, Turkey, Iran, Iraq, western India, southern Greece, Sicily (Fibiger 1990); Syria, Egypt ( Hacker 1990 ); Lebanon ( Hacker 2001 ); Israel ( Kravchenko et al. 2006 ).

**Distribution in Iran.** Sistan va Baluchestan ( Ebert and Hacker 2002 ); Hormozgan.

**Description.** Male ( Figure 4 ). Antennae slightly pectinated. Wingspan 32–43 mm; forewings light brown to gray, noctuid maculation complete, claviform and orbicular stigmata elongated, claviform stigma light brown surrounded by black line, orbicular stigma cream, reniform stigma elongated, surrounded by black line, cross lines distinguishable; hindwing pure white without discal spot. Female similar to male but larger than male; antennae filiform.

**Male genitalia** ( Figure 19 ). Valve elongated, distal one-third relatively narrow, slightly pointed, costal and ventral margin curved, clasper in moderate size and approximately slender. Aedeagus basally and terminally narrow, medially broader. Vesica narrow, long, nearly about five times as long as the aedeagus, basal swelling with a small bulge, scobinated bar short, apical swelling cylindrical.

**Female genitalia** ( Figure 32 ). Ovipositor of medium size, papilla anales pointed posterior and anterior apophyses in moderate size, posterior apophyses longer than anterior ones; appendix bursae, curved, very long, more than three times longer than corpus bursae, both drop-shaped.

**Bionomy.** Univoltine. Late winter or early spring flyers, from October to April. Species attracted to light ( Kravchenko et al. 2006 ). Widespread in desert and semi-desert habitats. The early stages and host plants are unknown.

**Taxonomic notes.** It can be confused with *A. puta,* but the basal area of the forewing in *A. puta* is dark colored, as is that of *A. syricola.* The aedeagus in *A. puta* is absolutely tubular and the papilla anales are short and not acute. *A. herzogi* also might be confused with *A. sardzeana,* but the forewings of *A. sardzeana* are light yellowish brown and its clasper is pointed.

**Material examined.** 1 *♂,* 1 **♀** , Iran. Prov. Hormozgan, Bandarabbas, Genou M., 500 m., 27°26'40"N 56°17'48"E, 05.03.2009. leg. A. Shirvani **.**

### *Agrotis puta* (Hübner, 1803)

*Noctua puta* Hübner, [1803], *Sammlung Europäischer Schmetterlinge***4** : pl. 52, Figure 255. L.t: Europe.

[Synonymy: *radius* (Haworth, 1803); *radiola* Stephens, 1829]

**General distribution.** Southwest of Palaearctic, including Southern Europe, North Africa, Turkey, Levante, Arabian Peninsula, Iraq, Iran, Turkmenistan ( Fibiger 1990 ); Cyprus ( Fibiger et al. 1999 ); Israel ( Kravchenko et al. 2007 ); Austria, France, Belgium, Southurn Germany, Hungary, Denmark, Poland, Britain ( Bond and Gittings 2008 ).

**Distribution in Iran.** Fars, Boushehr ( Ebert and Hacker 2002 ).

**Description.** Male ( Figure 5 ). Antennae pectinated. Wingspan 28–32 mm; forewing whitish cream, reniform stigma black mixed with dark brown, orbicular stigma whitish, centered with dark spot, claviform stigma light brown surrounded by black band, cross lines almost obsolescent, basal area dark, terminal area medially with conspicuous brown patch; hindwings white to cream. In female, antennae filiform; forewing darker than male.

**Male genitalia** ( Figure 20 ). Valve elongated, costal margin and ventral margin relatively straight, cucullus nearly slender apically, clasper sickle-like. Aedeagus medially broader; vesica more than five times longer than the aedeagus, basal diverticulum nearly rectangular, scobinated bar bent, with some wrinkled grooves around.

**Female genitalia.** Described by Fibiger (1997 , Figure 67). Ovipositor small, papilla anales short, hairy, apophyses of moderate sizes; ductus bursae slender, slightly sclerotized; both the corpus bursae and appendix bursae drop-shaped terminally; appendix bursae twice as long as the corpus one.

**Bionomy.** Multivoltine, with two or three generations in a year ( Fibiger 1990 ), bivoltine in central Europe ( Kravchenko et al. 2007 ). Adults appear from September to May (Fibiger 1990) and May to October in central Europe ( Kravchenko et al. 2006 ). This species mainly inhabits open areas. The larvae are nourished by herbaceous and short-term growth plants and have been described by several authors.

**Taxonomic notes.** This species is externally similar to *A. syricola* , but the whitish cream forewing and the darker basal zone with conspicuous brown patch of medio-terminal area are characteristics of *A. puta.*

**Remarks:** So far, the presence of *A. syricola* in Iran has not been confirmed.

**Material examined.** 1 ♂, Yugoslavia, Macedonia, Strumica, 23.9.1973, light trap.

### *Agrotis sardzeana* Brandt, 1941

*Agrotis sardzeana* Brandt, 1941 , *Mitt. Münch. Ent. Ges* . **31** : 840. L.t.: Iran, Sardza, Bandar Chabahar.

**General distribution.S** outhwest to south of the Palaearctic region, toward Iran. Oman ( Wiltshire 1975 ); Egypt, Iraq ( Hacker 1990 ); Pakistan, India, Israel ( Brandt 1941 ; Kravchenko et al. 2006 ); Levante ( Hacker 2001 ); Turkey ( Kemal et al. 2007 ).

**Distribution in Iran.** Kerman, Lorestan, Sistan va Baluchestan ( Ebert and Hacker 2002 ); Golestan ( Wieser and Stangelmaier 2005 ).

**Description.** Male ( Figure 6 ). Antennae pectinated. Wingspan 34–42 mm; general color of forewings gray to brown, noctuid maculation complete, orbicular stigma elongated, white, reniform stigma connected to orbicular stigma, both centered by black, claviform stigma dark brown, antemedian and postmedian lines slightly indeterminate; hindwing white. Female resembles to the male, antennae filiform.

**Male genitalia** ( Figure 21 ). Valve elongated, distally one-third narrow, broader at the middle, costal margin slightly arched, clasper short, sharp, and bent. Aedeagus tubular; vesica tubular, very long, more than five times as long as the aedeagus, basal swelling irregular, scobinated bar long.

**Female genitalia** ( Figure 33 ). Ovipositor short, posterior apophyses longer than anterior ones; appendix bursae twice as long as the corpus bursae, with torsion.

**Bionomy.** Univoltine. Adults are active in mid-autumn and usually live in arid and semiarid habitats but sometimes exist in shrubs and forest vegetation. The early stages and host plants are undescribed.

**Taxonomic notes** . Imagines are similar to *A. herzogi,* but *A. sardzeana* have white orbicular stigma and light brown areas displayed on the forewings.

**Material examined.** 10 *♂,* Iran. Prov. Zahedan, L. South, 1400 m., 01.11.2004 and 03.10.2006; Prov. Kerman, Lalezar, 3200 m., 29°27'33"N 56°48'33"E, 25.06.2009. leg. M. Shoghali; Prov. Sistan va Baluchestan, Zahedan, 1550 m., 29°21'53"N 60°47'50"E, 17.11.2009. leg. E. Kazemi; Prov. Sistan va Baluchestan, Nosratabad, 1370 m., 29°40'37"N 59°53'15"E, 19.11.2009. leg. E. Kazemi.

## The *trux* species-group

### Agrotis exclamationis (L., 1758)

*Phalaena Noctua exclamationis* L., 1758, *Systema Naturae* ( *End 10* ) **1** : 515. L.t.: Europe

[Synonymy: *picea* (Haworth, 1809); *plaga* Stephens, 1835]

**General distribution.** Palaearctic region. Widespread through the entire Palaearctic region except Iceland. In North Africa and the Near and Middle East, this species is limited in semi-desert and desert zones ( Kravchenko et al. 2006 ). In Iraq ( Hacker 1990 ); Mongolia ( Gyulai and Ronkay 1999 ); Lebanon, Caucasus, Russia, Turkey, Armenia, Afghanistan, Turkmenistan ( Ivinskis and Miatleuski 1999 ); Cyprus ( Hacker 2001 ).

**Distribution in Iran.** Tehran, Mazandaran, Azarbayjan-e-Sharghi, Kordestan, Ardabil, Fars, Hormozgan, Esfahan ( Hacker and Kautt 1999 ); Azarbayjan-e-gharbi, Elburz Mountain ( Ebert and Hacker 2002 ); Sistan va Baluchestan, Semnan, Kerman, Markazi.

**Description.** Male ( Figure 7 ). Antennae weakly pectinated. Patagia with black line. Wingspan 36–39 mm; forewings bright to dark brown, forewings ground color, almost unicolor except the terminal area is darker, noctuid maculation present, reniform and claviform stigmata distinct, black, orbicular stigma as ground color in some specimens a little darker, antemedian and postmedian lines more or less distinct, sometimes weak, the former more distinct; hindwings white to light cream. Female similar to male, antennae filiform.

**Male genitalia** ( Figure 22 ). Valve long, acute, costal margin slightly arched, clasper slightly pointed. Vesica long, more than five times as long as the aedeagus, basal swelling elliptical, broad, with basal bulge, scobinated bar long, turned, apical swelling distinct.

**Female genitalia.** Described by Fibiger (1997 , Figure 77). Ovipositor short, posterior apophyses a little longer than anterior ones; ductus bursae short, narrower at distal half, sclerotized bands present; appendix bursae and corpus bursae tubular, globularly widened; appendix bursae long, curved, about three times as long as the corpus bursae.

**Bionomy.** Multivoltine. The reported flight period in Israel is November ( Kravchenko et al. 2006 ), but it has been recorded in May to July in some countries, like Ireland ( Bond and Gittings 2008 ). In Iran, it has been collected in May to October and usually occurs in open spaces, especially in farming lands, in all altitudes. Adults are attracted to light and feed on nectar of flowers. Larvae are known as a serious pest of agricultural crops, feeding on roots of herbaceous plants, specifically plants from the Gramineae family.

**Taxonomic notes.** It might be confused with *A. clavis,* but the dark line on patagia is missing in *A. clavis.* Also, the black dart-shaped claviform stigma is a distinctive feature of *A. exclamationis.*

**Material examined.** 5 *♂,* Iran. Prov. Kerman, Honouj, 3200 m., 28.05.2006; Prov. Kerman, Bondar, 2300 m., 06.09.2006 and 05.10.2006; Prov. Kerman, Chatroud, Paye Sib, 2320 m., 30°37'33"N 57°02'34"E, 21.05.2009.leg. M. Shoghali; Prov. Kerman, Lalezar, 3200 m., 29°27'33"N 56°48'33"E, 25.06.2009. leg. M. Shoghali.

### *Agrotis trux* (Hübner, [1824])

*Noctua trux* Hübner, [1824], *Sammlung Europäischer Schmetterlinge***4** : pl. 155, Fig. 723, 725, pl. 163, Figure 770. L.t.: Europe.

[Synonymy: *lenticulosa* Duponchel, 1826; *lunigera* Stephens, 1829; *terranea* Freyer, 1831; *amasina* Staudinger, 1901; *subalba* Corti & Draudt, 1933; *adolfi* Corti & Draudt, 1933]

**General distribution.** Palaearctic region, including Britain, France, Ireland, Israel, Turkey, Iraq, North Africa, Arabian Peninsula ( Fibiger 1990 ); Syria, Lebanon, Egypt ( Hacker 1990 ); Cyprus ( Fibiger et al. 1999 ); Jordan ( Hacker 2001 ).

**Distribution in Iran.** Guilan, Elburz Mountains ( Ebert and Hacker 2002 ); Sistan va Baluchestan, Kerman, Hormozgan.

**Description.** Male ( Figure 8 ). Antennae pectinated. Wingspan 35–38 mm; forewings beige, noctuid maculation distinct, orbicular stigma surrounded by black line, claviform stigma small, slightly elongated, encircled with black line as well as reniform stigma, cross lines identifiable but antemedian and postmedian lines obsolescent, costal margin apically with dark spot; hindwings white to bright cream. Female as male, antennae filiform.

**Male genitalia** ( Figure 23 ). Valve elongate, costal margin medially arched, ventral margin slightly convex medially, valve slightly pointed, clasper sickle form. Aedeagus broader medially; vesica long, five times longer than the aedeagus, basal swelling small, pointed, apical swelling large, well defined, two times broader than the vesica.

**Female genitalia.** Described by Fibiger (1997 , Figure 75). Ovipositor small, papilla anales rectangular, apophyses slender with equal length; ostium bursae strongly sclerotized; ductus bursae long; appendix bursae three times as long as the corpus bursae, curved, both of them drop-shaped terminally.

**Bionomy.** Probably multivoltine. Flight period in October, March and June in Iran. July to mid-August in Ireland, July and August in Britain ( Bond and Gittings 2008 ), September to April in Israel ( Kravchenko et al. 2006 ). The adults are attracted to light, sugar, and flowers ( Fibiger 1990 ) and inhabit various areas. The larvae feed on grassy plants and have been described by several authors.

**Taxonomic notes.** Its diagnostic characters were discussed under *A. segetum.*

**Material examined.** 11 *♂,* Iran. Prov. Hormozgan, North of Bandar Abbas, Sirooye, 12.11.2005. leg. A. Shirvani; Prov. Sistan va Baluchestan, Taftan mt., 2600 m., 28°36'328"N 61°04'767"E, 2.11.2006. leg. A. Shirvani; Prov. Kerman, Jiroft, Dalfard, 28°59'06"N 57°38'32"E, 25.10.2008. leg. M. Shoghali; Prov. Kerman, Kouhbanan, km 5, Yazd Roaded, 31°27'52"N 56°16'45"E, leg. M. Shoghali; Prov. Kerman, Khabr, 2073 m., 28°49'02"N 56°20'02"E, leg. M. Shoghali; Prov. Sistan va Baluchestan, Khash road, Anari junction, 05.10.2010. leg. E. Kazemi.

### *Agrotis ipsilon* (Hufnagel, 1766)

*Phalaena ipsilon* Hufnagel, 1766, *Berlin. Mag.***3** : 416. L.t: Germany, Berlin district.

[Synonymy: *ypsilon* misspelling; *suffusa* (Denis & Schiffermüller, 1775); *spinula* (Esper, 1786); *spinifera* (Villers, 1789); *spinula* (Donovan, 1891)]

**General distribution.** Cosmopolitan.

**Distribution in Iran.** Azarbayjan-e-Sharghi, Tehran, Mazandaran, Kordestan, Fars, Lorestan ( Hacker and Kautt 1999 ); Hormozgan, Bushehr, Elburz Mountains ( Ebert and Hacker 2002 ); Kerman, Sistan va Baluchestan, Khorasan, Golestan, Guilan, Azarbayjan-e-gharbi, Zanjan, Semnan, Yazd, Qum, Markazi, Ardabil, Kermanshah, Ilam, Esfahan, Hame-dan.

**Description.** Male ( Figure 9 ). Antennae pectinated. Wingspan 36–43 mm; forewings light brown to dark brown, noctuid maculation complete, reniform and orbicular stigmata centered with black, a black dart shaped patch present in the ostium of the reniform stigma as well as the two small dart shaped patches in the subterminal area, these features are missing in other species of the genus *Agrotis* . Black band presented between the reniform and orbicular stigmata, terminal and subterminal areas bright; hindwing white and crystalline but darker in terminal area. Female as male, antenna filiform.

**Male genitalia** ( Figure 24 ). Valve elongated, basal one-third narrow, widened; clasper pointed. Aedeagus broader basally; vesica elongated, tubular, with small sclerotized grooves, six times as long as the aedeagus, basal swelling similar to cylinder, everted laterally, scobinated bar long, apical swelling weakly broader than the vesical tube.

**Female genitalia** ( Figure 34 ). Ovipositor small, both the apophyses short, posterior apophyses longer than the anterior ones; ductus bursae in the same length of the ovipositor, appendix bursae tubular, less than one-half times as long as the corpus bursae; corpus bursae pyriform.

**Bionomy.** Usually multivoltine. Active throughout the year throughout Iran. Moths attracted to flowers, light, and sugar ( Fibiger 1990 ) with heavy migration. Larvae feed on various plants belonging to many families and are one of the most important pests on herbaceous plants, feeding on the roots.

**Taxonomic notes.** Dart-shaped patches on the forewing are the most diagnostic features of this species among its other congeners.

**Material Examined.** 8 *♂,* 1 **♀** , Iran. Prov. Hormozgan, 3.11.2005. leg. A. Shirvani; Prov. Kerman, Kuhbanan, 2700 m., 31°23'09"N 56°13'24"E, 21.09.2009, 23.03.2009 and 22.04.2009. leg. M. Shoghali; Prov. Kerman, Chatroud, Paye Sib, 2300 m., 30°37'33"N 57°02'34"E, 04.10.2008. leg. M. Shoghali; Prov. Kerman, Sirch Tunnel, 2726 m., 30°09'20"N 57°24'17"E, 27.03.2009. leg. M. Shoghali; Prov. Kerman, Sirch Tunnel, 2726 m., 30°08'56"N 57°24'32"E, 27.03.2009. leg. M. Shoghali; Prov. Kerman, Sirjan-Shiraz road, 60 km Sirjan, 29°25'54"N 55°07'38"E, 30.04.2009. leg. Z. Bidar; Prov. Kerman, Lalezar, 3200 m., 29°27'33"N 56°48'33"E, 25.06.2009. leg. M. Shoghali.

## The *bigramma* species-group

### *Agrotis obesa scytha* Alphéraky, 1889

*Agrotis obesa* var. *scytha* Alphéraky, 1889, *In Romanoff Mem. Lep.***5** : 143. L.t: Caucasus.

[Synonymy: *lipara* Rambur, 1848 (Lerautm 1980); *nivea* Caradja, 1932]

**General Distribution.** Palaearctic region. This species has been recorded from Europe, Southern former Soviet Union, Turkey, Syria, Iraq, Iran ( Fibiger 1990 ); Turkmenistan (Ivinskis and Miatleuski 1999); Kazakhstan, Kirghisia, Uzbekistan ( Hacker 2001 ); Western China, North Africa, Israel, Lebanon, Jordan ( Kravchenko et al. 2007 ).

**Distribution in Iran.** Gorgan, Khorasan ( Wieser and Stangelmaier 2005 ); Tehran, Elburz Mountains, Guilan ( Ebert and Hacker 2002 ); Kerman.

**Description.** Male ( Figure 10 ). Antennae strongly pectinated. Wingspan 36-45 mm; forewings bright brown to bright gray, all three spots distinct, reniform and claviform stigmata darker than forewing ground color, a dark patch between reniform and orbicular stigmata, antemedian and postmedian lines not distinct, subterminal line complete, white streak extended from the upper edge of reniform stigma to the cilia, veins distinct surrounded by white scales; hindwing bright, sometimes the outer margin light brownish. Female as male but antennae filiform and outer margin of the hindwing gray to brown.

**Male genitalia** ( Figure 25 ). Valve slightly arched at the costal margin, clasper sickle-shaped. Aedeagus nearly tubular; vesica tubular, four times longer than the aedeagus, basal swelling with almost large rounded dorsal hump, scobinated bar relatively short.

**Female genitalia.** Described by Fibiger (1997 , Figure 80). Ovipositor large, posterior and anterior apophyses in the same length, posterior apophyses slender; ductus bursae nearly in the same length of ovipositor; appendix bursae short, slightly longer than corpus bursae; corpus bursae elliptical.

**Bionomy.** Univoltine, flying generally from August to October, adults attracted to sugar and light. In Israel, a univoltine autumnal species flying from September to October ( Kravchenko et al. 2006 ). This species is mostly found in open habitats along with extending vegetation. The early stages feed on sprouts and aerial parts of fresh plants.

**Taxonomic notes.** The imagines of *A. obesa* are externally similar to those of *A. bifurca,* but veins on forewings are well outlined by light scales, and the outer half of the hindwing and its fringes are dark brown.

**Material Examined.** 18 **♂** , Iran. Prov. Kerman, L. Bondar, 2300 m., 05.10.2004; Prov. Kerman, L. Sirch, 2400 m., 06.10.2004; Prov. Kerman, Chatroud, Paye Sib, 2320 m., 30°37'33"N 57°02'34"E, 13.10.2009. leg. M. Shoghali; Prov. Kerman, Sirch Tunnel, 2700m., 30°08'57"N 57°24'32"E, 15.10.2009. leg. M. Shoghali.

## *Agrotis bifurca grossi* Hacker & Kuhna, 1986

*Agrotis grossi* Hacker & Kuhna, 1986, *Nota Lepid.***9** : 180. L.t: Turkey.

**General distribution.** Russia, Turkey ( Hacker 1990 ); Turkmenistan.

**Distribution in Iran.** Khorasan ( Ebert and Hacker 2002 ).

**Description.** Male ( Figure 11 ). Antennae pectinated. Wingspan 36-44 mm; forewings light brown to cream, noctuid maculation distinguishable, reniform stigma darker than the forewing ground color, orbicular stigma cream, claviform stigma dark brown surrounded by black band, a dark brown band between the reniform and orbicular stigmata, as color as the claviform stigma, an irregular dark brown band present on outer margin of the forewings; hindwing white to cream, discal spot present as a small gray spot. Female not available.

**Male genitalia** ( Figure 26 ). Distal one third of valve narrow rounded apically, clasper short, sickle form. Aedeagus and vesica tubular; more than three times as long as the aedeagus, basal swelling dorsally armed with a small rounded diverticulum, scobinated bar in moderate size; vesica tube tapering.

**Bionomy.** Univoltine. This species was collected in October. Attracted to artificial light. The early stages and host plants are unknown in Iran.

**Taxonomic notes.** It has been diagnosed with its closest relative, *A* . *obesa* , above.

**Material examined.** 1 ♂, USSR. Turkmenia, Kopet-Dagh Mountains, 2200 m., Dushak, 37°57′N 57°54′E, 01-02.10.1991. leg. Podlussany, L. Ronkay & Z. Varga.

## *Agrotis bigramma* (Esper, [1790])

*Phalaena Noctua bigramma* Esper, [1790], *Die Schmett. In Abb. Nach. Der Natur***4** : 490, L. t.: Germany.

[Synonymy: *crassa* (Hübner, [1803]); *con-spicua* Hübner, [1824]; *lata* Treitschke, 1834; *golickei* Ershov, 1872]

**General distribution.** Palaearctic region. *A. bigramma* has been reported from Europe ( Fibiger 1990 ); Egypt, Iraq, Turkey, Afghanistan, Caucasus, Lebanon ( Hacker 1990 ); Cyprus ( Fibiger et al. 1999 ); the Altai, China, Morocco, western Algeria, the Levante ( Hacker 2001 ); Israel ( Kravchenko et al. 2007 ).

**Distribution in Iran.** Tehran, Guilan, Elburz Mountain ( Ebert and Hacker 2002 ); Golestan ( Wieser and Stangelmaier 2005 ).

**Description.** Male ( Figure 12 ). Antennae strongly pectinated, being weak toward the tip. Wingspan 36-50 mm; forewings pale brown to dark brown, noctuid maculation complete, reniform and claviform stigmata nearly dark brown, orbicular stigma cream, antemedian and postmedian lines distinct, crenate; hindwing pure white. Female as male, antennae filiform.

**Male genitalia** ( Figure 27 ). Valve slightly arched at the costal margin, cucullus tapering, clasper sickle form, long. Aedeagus tubular; vesica long, more than four times longer than aedeagus, basal swelling bears strong basal diverticulum, sclerotized stripe short, apical swelling only slightly wider than vesica tube.

**Female genitalia.** Described by Fibiger (1997 , Figure 79). Ovipositor long, gonapophyses very long, anterior apophyses shorter than posterior ones, reaching the base of appendix bursae; ostium bursae membranous; ductus bursae medium size, distal one-third constricted; appendix bursae long and curved, tubular, twice longer than corpus sac; corpus bursae long, widened.

**Bionomy.** Univoltine in Israel and Europe ( Fibiger 1990 ; Kravchenko et al. 2007 ). Adults are on the wing in September to December in Israel and in August and September in Europe. Imagines attracted to flowers, sugar, and artificial light. Occurs in steppe regions with sparse vegetation ( Hacker 2001 ). Larvae feed on Poaceae species and low density vegetation ( Hacker 2001 ).

**Taxonomic notes.** This species externally resembles *A. obese scytha,* but the main diagnostic features presented in *A. bigramma* are as follows: noctuid maculation are brighter without dark band surrounded by reniform and orbicular stigmata; Longitudinal veins not covered with white scales; crenate antemedian and postmedian lines distinct; hindwings pure white; in the male genitalia, basal swelling with strong basal diverticulum.

**Material Examined.** 1 ♂ Yugoslavia. Mazedonia, Strumica, 2.9.1971, light trap.

## The *spinifera* species-group

### *Agrotis spinifera* (Hübner, [1808])

*Noctua spinifera* Hübner, [1803], *Sammlung Europäischer Schmetterlinge***4** : pl. 83, Figure 389. L.t: Europe. [Synonymy: *biconica* Kollar, 1844]

**General distribution.** Palaearctic region, Ethiopian and Oriental regions ( Kravchenko et al. 2006 ).

**Distribution in Iran.** Fars, Lorestan ( Hacker and Kautt 1999 ); Golestan ( Wieser and Stangelmaier 2005 ); Tehran, Elburz Mountains, Guilan, Mazandaran, Sistan va Baluchestan, Bushehr, Hormozgan ( Ebert and Hacker 2002 ); Kerman, Semnan.

**Description.** Male ( Figure 13 ). Antennae weakly pectinated. Wingspan 28-40 mm; forewings brown to gray, three stigmata complete, orbicular stigma elongated, claviform stigma sharply defined, pointed, pure black, a black band present between the reniform and orbicular stigmata, antemedian and postmedian lines missing, subterminal line distinct as an incomplete waved line, terminal line distinct; hindwings white to bright cream. Female as male, antennae filiform.

**Male genitalia** ( Figure 28 ). Valve of moderate size, costal margin approximately straight, ventral margins arched, clasper short and thickened. Aedeagus broader basally, two subbasal spine fields at the apex of aedeagus; vesica tubular, four times as long as the aedeagus, basal swelling pyriform, scobinated bar of medium size, spiny.

**Female genitalia** ( Figure 35 ). Ovipositor relatively large, posterior apophyses a little longer than anterior ones; ostium bursae sclerotized. Both appendix and corpus bursae tubular; corpus bursae medially tubular; appendix bursae slightly longer than the corpus bursae.

**Bionomy.** Multivoltine. Active throughout the year ( Kravchenko et al. 2006 ). Attracted to light, and appears in various geographical regions with various altitudes. The early stages have been described by several authors. The caterpillars feed on the leaves and roots of herbaceous plants, especially on Poaceae family.

**Taxonomic notes.** This species can be distinguished from its other Iranian congeners by a very long, black claviform stigma and elongated orbicular stigma.

**Material Examined.** 1 *♂* 1 **♀** , Iran. Kerman, Shahdad, 400 m., 30°25'08"N 57°4246"E, 23.02.2010. leg. M. Shoghali; Prov. Kerman, Khabr, 2073 m., 28°49'02"N 56°20'02"E, 20.10.2010. leg. M. Shoghali.

### *Agrotis lasserrei* (Oberthür, 1881)

*Luperina lasserreiOberth* ür *,* 1881, *Etudes Ent. Comp.***6** : 86. L.t: Algeria.

[Synonymy: *unctus* Christoph, 1887; *sabura* Mabille, *1888; ptolemaidea* Turati, 1924]

**General distribution.** South of Palaearctic region especially in droughty places ( Kravchenko et al. 2006 ); Lebanon, Syria, Israel, Iraq, Jordan, Arabian Peninsula, Egypt ( Hacker 1990 ); In Europe, in Spain and Malta; from the West to Mauretania, West Sahara in North Africa and the Canary Isles, from the East to Turkmenistan and Iran ( Hacker 2001 ).

**Distribution in Iran.** Golestan, Khorasan ( Wieser and Stangelmaier 2005 ).

**Description.** Male ( Figure 14 ). Antennae strongly pectinated. Wingspan 29–33 mm; forewing general color grayish brown, noctuid maculation complete, reniform and orbicular stigmata surrounded by white lines, claviform stigma brown, transverse lines distinct; hindwings white to light cream, discal spot weak. Female as male, antennae filiform.

**Male genitalia.** Described by Fibiger (1997 , Figure 83). Costal margin straight, clasper sickle form. Aedeagus tubular; vesica short, tapering, twice as long as the aedeagus, basally broad with swelling, scobinated bar very short, its width ceasing toward the end of vesica.

**Female genitalia.** Described by Fibiger (1997 , Figure 83).Ovipositor small, papilla anales stumpy, posterior and anterior apophyses short; ostium bursae fairly sclerotized; ductus bursae and corpus bursae short, rugu-lose, swelling basally, medially less broad, widened; appendix bursae small.

**Bionomy.** Univoltine, with autumnal flight period ( Kravchenko et al. 2006 ). This species is widespread in arid and semiarid regions. Early stages and host plants are as yet unknown.

**Taxonomic notes.** None of the other Iranian *Agrotis* species looks like *A. lasserrei* .

**Material examined.** 1 ♂, USSR. Turkmenia, Kara-Kum desert, 42 km N of Ashkhabad, 100 m., 38°21'N 58°33'E, 15.10.1991. leg. A. Podlussany, L. Ronkay & Z. Varga.

### *Agrotis benigna* (Corti, 1926)

*Cladocerotis benigna,* 1926, *Dt. Ent. Z. Iris***39** : 231. L.t: Syria.

**General distribution.** Russia, Turkey, Iraq ( Hacker 1990 ).

**Distribution in Iran.** Tehran, Elburz Mountains ( Ebert and Hacker 2002 ); Kerman, Sistan va Baluchestan.

**Description.** Male ( Figure 15 ). Antennae strongly pectinated. Wingspan 34-38 mm; general color gray-cream mixed with light brown, noctuid maculation fade, transverse lines missing; hindwing white, discal spot present.

**Male genitalia** ( Figure 29 ). Valve pointed, clasper thickened. Aedeagus tubular; vesica short, simple, tubular, tapering, basal swelling distinct, scobinated bar in moderate size.

**Bionomy.** Univoltine. This species flies in October and is attracted to light. The early stages and host plants are yet undisclosed.

**Taxonomic notes.** The imagines of *A. benigna* are similar to those of *A. psammocharis,* but some differences between them have been discussed in the section pertaining to *A. psammocharis.*

**Material examined.** 9 *♂,* Iran. Prov. Kerman, Sirch Tunnel, 2700m., 30°08'57"N 57°24'32"E, 06.10.2008. leg. M. Shoghali; Prov. Sistan va Baluchestan, Nosratabad, 1370 m., 29°49'37"N 59°53'15'E, 28.10.2009. leg. E. Kazemi; Prov. Sistan va Baluchestan, Zahedan, 1550 m., 29°21'53"N 60°47'50"E, 17.11.2009. leg. E. Kazemi.

### *Agrotis psammocharis* Boursin, 1950

*Agrotis psammocharis* Boursin, 1950, Archiv f. Zool. **1** : 355. L.t.: Iran, Elburz Mountains.

**General distribution.** Iran, Syria, Lebanon, Israel, Jordan, Turkmenistan ( Kravchenko et al. 2007 ).

**Distribution in Iran.** Tehran ( Hacker 1990 ); Karaj, Elburz Mountains ( Kravchenko et al. 2007 ); Kerman, Sistan va Balouchetan.

**Description.** Male ( Figure 16 ). Antennae strongly pectinated. Wingspan 33–41 mm; forewing ground color light brown to gray, reniform and orbicular stigmata present as a two pale gray signs, claviform stigma absent, antemedian and postmedian lines distinguishable; hindwing white with an elongated discal spot. Female as male, antennae filiform.

**Male genitalia** ( Figure 30 ). Costal and ventral margin of the valve straight, cucullus rounded; clasper almost sharp. Aedeagus tubular, nearly as long as the vesica; vesica short, basal swelling nearly two times as broad as the aedeagus, scobinated belt in moderate size, vesica narrower to the end.

**Female genitalia.** Female specimens were not collected in this survey.

**Bionomy.** Univoltine. Adults commonly fly in fall. They are attracted to light, sugar, and flowers and are present in areas with poor vegetation and dry climate. The early stages and host plants are unknown.

**Taxonomic notes.** External characteristics of this species are close to *A. benigna* but can be recognized by absence of claviform stigma in *A. psammocharis* and in the male genitalia by thickened clasper and longer vesica, which is more than three times longer than aedeagus in the latter species.

**Material examined.** 11 *♂,* Iran. Prov. Kerman, Kouhpaye, Dehlolo, 2000 m., 30°28'59"N 56°20'02"E, 02.10.2008. leg. M. Shoghali; Prov. Kerman, Sirch, 2726 m., 30°08'56"N 57°24'32"E, 06.10.2008 and 15.10.2009 leg. M. Shoghali; Prov. Kerman, 25 km S Kerman, Joupar, 30°04'63"N 57°05'58"E, 23.10.2008. leg. Z. Bidar; Prov. Kerman, 50 km S Sirjan, Godar, 29°46'34"N 56°55'02"E, 29.10.2008. leg. Z. Bidar; Prov. Kerman, Chatroud, Paye Sib, 2320 m.,30°37'33"N 57°02'34"E, 13.10.2009. leg. M.A. Shoghali.

## References

[R1] Bond KGM Gittings T . 2008 . Database of Irish Lepidoptera. 1- Macrohabitats, microsites and traits of Noctuidae and butterflies . In: *Irish wildlife manuals,No* . 35 . National Parks and Wildlife Service, Department of the Environment, Heritage and Local Government, Dublin, Ireland.

[R2] Brandt W . 1941 . Beitrag zur Lepidopteran-Fauna von Iran (3). Neue Agrotiden, nebst faunen verzeichnissen . Mitteilungen der Münchener entomologischen Gesellschaft 31 : 835-863.

[R3] Ebert G Hacker H . 2002 . Beitrag zur Fauna der Noctuidae des Iran: Verzeichnis der Bestände im Staatlichen Museum für Naturkunde Karlsruhe, taxonomische Bemerkungen und Beschreibung neuer Taxa (Noctuidae, Lepidoptera) . Esperiana Buchreihe zur Entomologie 9 : 1-606, 25 Taf.

[R4] Fibiger M . 1990 . Noctuinae I . Noctuidae Europaeae Vol. 1 . Entomological Press, Søro, Denmark.

[R5] Fibiger M . 1993 . Noctuinae II . Noctuidae Europaeae Vol. 2 . Entomological Press, Søro, Denmark.

[R6] Fibiger M . 1997 . Noctuinae III . Noctuidae Europaeae Vol. 3 . Entomological Press, Søro, Denmark.

[R7] Fibiger M Nilsson D Svendsen P . 1999 . Contribution to the Noctuidae fauna of Cyprus, with descriptions of four new species, six new subspecies, and reports of 55 species not previously found on Cyprus (Lepidoptera, Noctuidae) . Esperiana Buchreihe zur Entomologie 7 : 639-667.

[R8] Gyulai P Ronkay L . 1999 . The Noctuidae (Lepidoptera) material collected by two Hungarian expeditions to Mongolia in 1996 and 1997 . Esperiana Buchreihe zur Entomologie 7 : 687-713.

[R9] Hacker H . 1990 . Die Noctuidae Vorderasiens (Lepidoptera) . Systematische List mit einer Übersicht über die Verbreitung unter besondere Berücksichtigung der fauna der Türkei (einschließlich der Nachbargebiete Balkan, Südrußland, Westturkestan, Arabische Halbinsel, Ägypten). Neue Entomologische Nachrichten 27 : 1-707.

[R10] Hacker H . 2001 . Fauna of the Nolidae and Noctuidae of the Levante with descriptions and taxonomic notes (Lepidoptera, Noctuoidea). Appendix: Revision of the genus *Clytie HBN* . Esperiana Buchreihe zur Entomologie 8 : 7-398.

[R11] Hacker H Kautt P . 1999 . Noctuoidea aus dem Iran, gesammelt 1997 von A. Hofmann und P. Kautt (Insecta, Lepidoptera) . Esperiana Buchreihe zur Entomologie 7 : 473-484.

[R12] Ivinskis P Miatleuski J . 1999 . Data on Noctuidae (Lepidoptera) of Turkmenistan . Acta Zoologica Lituanica 9(1): 201-208.

[R13] Kemal M Seven S Koçak AO . 2007 . List of the Irano-Anatolian Noctuidae with some faunal and zoogeographical remarks based upon the Info-System of the Cesa (Lepidoptera) . PRIMUS 9 : 1-89.

[R14] Koçak AO Kemal M Seven S Özkol H Kayci L . 2008 . Noctuidae Fauna of the Caucasus Region (Lepidoptera) . PRIMUS 11 : 1-128.

[R15] Kravchenko VD Fibiger M Mooser J Müller GC . 2006 . The Noctuinae of Israel (Lepidoptera: Noctuidae) . SHILAP Revista de Lepidopterología 34(136): 353-370.

[R16] Kravchenko VD Fibiger M Hausmann A Müller GC . 2007 . Vol. 2, Noctuidae. In: Müller GC, Kravchenko VD, Hausmann A, Speidel W, Mooser J, Witt TJ, Editors . The Lepidoptera of Israel . pp. 1-320. Pensoft Publishers.

[R17] Lafontaine JD Schmidt BCh . 2010 . Annotated check list of the Noctuoidea (Insecta, Lepidoptera) of North America north of Mexico . ZooKeys 40 : 1-239. 10.3897/zookeys.149.1805 PMC3234417 22207802

[R18] Michael GP . 2006 . The Noctuinae (Lepidoptera: Noctuidae) of Great Smoky Mountains National Park, U.S.A . Zootaxa 1215 : 1-95.

[R19] Varga Z . 1996 . Biogeography and evolution of oreal Lepidoptera in the Palaearctic . Acta Zoologica Academiae Scientiarum Hungarica 42(4): 289-330.

[R20] Wieser VCh Stangelmaier G . 2005 . Zwischenergebnisse einer lepidopterologischen Forschungsreise in den NordIran, Oktober 2003 (Insecta: Lepidoptera) . Carinthia II 195/ 115 : 659-674.

[R21] Wiltshire EP . 1994 . Arabian Lepidoptera: a Supplement to the Catalogue of Saudi Arabian Macro-Heterocera . Fauna of Saudi Arabia 14 : 113-126.

[R22] Wiltshire EP . 1975 . Lepidoptera: Part II. A list of further Lepidoptera-Heterocera from Oman with remarks on their economic importance and descriptions of one new genus, four new species, and two new subspecies . The Journal of Oman Studies special report no. 1 : 155-178.

